# Notes and News

**Published:** 1892-10-29

**Authors:** 


### Notes and News.

Collected and Communicated.

The Irish National Consumptive Hospital scheme is in hot water. It is difficult to make out the case without hearing both sides, but the pity is in such a scheme that there should be "sides" at all. The Hon. Secretary and promoter of the hospital has resigned. She intimates that the cause of dispute is a religious one; that she desired that both Roman Catholic and Protestant patients should have equal religious privileges through the establishment of separate chapels in connection with the institution; that this has been opposed by the Catholics, and her right to be regarded as the founder of the hospital scheme ignored. We cannot help surmising that Miss Wynne rather overrates the injuries done to herself and to the "Church of Ireland," which we were unaware existed except in the lady's imagination.

There seems to be some misapprehension still about our first note on the Salisbury Infirmary. We have received a letter from an old Salisbury Probationer, for instance, which we do not publish, because it shows that there is prejudice in favour of one officer over another on the part of certain people, although between the two officers themselves there is not only no friction, but a real and cordial feeling of sympathy and respect. In these circumstances, having made a careful inspection of the Salisbury Infirmary a few weeks ago, we must once more emphatically state that its present condition is excellent, and that much of that excellence is due to the zeal and exceptional abilities of the present Matron, Miss Carvosso. Miss Carvosso's abilities entitle her to occupy a much more responsible post than her present one, and we look forward to the time when she will be selected for the Matronship of one of the great hospitals. We say this, although Miss Carvosso is an entire stranger to ourselves, and we only know her through her work, which has been remarkably well done. Surely there are sufficient independent Governors connected with the Salisbury Infirmary who possess the necessary amount of public spirit to secure their attendance at the next meeting of Governors in support of the Chairman and Committee who have wisely decided that Miss Carvosso has earned an increase of salary.

The new quarters of the Home for the Dying, known as the Friedenheim, are to be opened by the Duchess of Teck, accompanied by the Princess May, on November 7th. The new quarters are situated near the Swiss Cottage Station, and will accommodate fifty patients, male and female, together with the staff. This Home is the only one of its kind in London, and in a previous number a description of the institution has been given.

The admission of ladies as clinical students at the South Infirmary, Cork, which appeared to be an accomplished fact, is destined again to come under discussion. It appears that the constitution of the institution sets forth that no rule can be altered without a month's notice, and an intimation of the proposed change shall be sent to each trustee three days previous to the meeting which shall be held to consider it. Some of the trustees who hold the innovation as equivalent to a change of rules, require that this arrangement shall be adhered to, and a meeting to discuss once again the admission of lady students will be held in due course.

Another striking proof of the pernicious tendency of street alms was shown at Chester recently. This was the case of a poor crippled girl who was kept begging by her mother day by day, as she found it paid so much better to send her unfortunate child to excite public pity than to beg herself. The mother spent the earnings in drink. Beggars are astute enough to realise what pays best, and so those who give the careless penny as a salve for their momentary feeling of distress at the sight of want or suffering are incurring a mighty responsibility. At their door rests the blame that deformity and exposure of little children is a trade so paying that honest labour is frequently deserted on its account.

There are some spots in this great metropolis of ours, in which a hospital proves more especially a blessing. Amidst wretched tenements and the surroundings of poverty and dirt, it stands out as a beacon which points to higher and better things. Order, comfort, and cleanliness, those are amongst the most striking lessons it teaches, and of these the once-patient is the more constantly reminded if the example is constantly in view. The women learn the beauty, if not he sanitary, advantage of clean boards, and we have ourselves heard one patient remark to another that she should have her bit of carpet up, which was old and worn, and scour her boards once or twice a-week, and show the neighbours what a floor should look like when she got home. The men recognise the advantage of order, and we know of many instances in which offers of marriage have been made to nurses in various hospitals, in compliment to the profession, not to the individual. Order and cleanliness we are taught are next to godliness, and when a charity combines the attributes of a mission and hospital, it has a double sphere of usefulness. Such an institution is the Mildmay Mission Hospital, now erecting in those haunts of sickness and poverty, Bethnal Green and Shoreditch. For fifteen years a temporary building has existed, and proved of so great a benefit, that now a permanent structure is to succeed it. Many generous friends for the new undertaking have been found, and it is earnestly hoped that the remaining £3,000 required will be forthcoming to complete the excellent work.

